# Black-blood thrombus imaging (BTI): a contrast-free cardiovascular magnetic resonance approach for the diagnosis of non-acute deep vein thrombosis

**DOI:** 10.1186/s12968-016-0320-8

**Published:** 2017-01-18

**Authors:** Guoxi Xie, Hanwei Chen, Xueping He, Jianke Liang, Wei Deng, Zhuonan He, Yufeng Ye, Qi Yang, Xiaoming Bi, Xin Liu, Debiao Li, Zhaoyang Fan

**Affiliations:** 1 Lauterbur Research Center for Biomedical Imaging, Shenzhen Institutes of Advanced Technology, CAS, Guangdong, 518055 China; 2 Biomedical Imaging Research Institute, Cedars-Sinai Medical Center, Pacific Theatres Building, Suite 800, 8700 Beverly Blvd, Los Angeles, CA 90048 USA; 3 Department of Radiology, Guangzhou Panyu Central Hospital, Guangzhou, Guangdong 511400 China; 4 Guangzhou University of Chinese Medicine, Guangzhou, Guangdong 510405 China; 5 Department of Radiology, Xuanwu Hospital, Beijing, 100053 China; 6 MR R&D, Siemens Healthcare, Los Angeles, CA 90048 USA

**Keywords:** Cardiovascular Magnetic Resonance, Black-blood thrombus imaging, Deep vein thrombosis

## Abstract

**Background:**

﻿Deep vein thrombosis (DVT) is a common but elusive illness that can result in long-term disability or death. Accurate detection of thrombosis and assessment of its size and distribution are critical for treatment decision-making. In the present study, we sought to develop and evaluate a cardiovascular magnetic resonance (CMR) black-blood thrombus imaging (BTI) technique, based on delay alternating with nutation for tailored excitation black-blood preparation and variable flip angle turbo-spin-echo readout, for the diagnosis of non-acute DVT.﻿

**Methods:**

This prospective study was approved by institutional review board and informed consent obtained from all subjects. BTI was first conducted in 11 healthy subjects for parameter optimization and then conducted in 18 non-acute DVT patients to evaluate its diagnostic performance. Two clinically used CMR techniques, contrast-enhanced CMR venography (CE-MRV) and three dimensional magnetization prepared rapid acquisition gradient echo (MPRAGE), were also conducted in all patients for comparison. All images obtained from patients were analyzed on a per-segment basis. Using the consensus diagnosis of CE-MRV as the reference, the sensitivity (SE), specificity (SP), positive and negative predictive values (PPV and NPV), and accuracy (ACC) of BTI and MPRAGE as well as their diagnostic agreement with CE-MRV were calculated. Besides, diagnostic confidence and interreader diagnostic agreement were evaluated for all three techniques.

**Results:**

BTI with optimized parameters effectively nulled the venous blood flow signal and allowed directly visualizing the thrombus within the black-blood lumen. Higher SE (90.4% vs 67.6%), SP (99.0% vs. 97.4%), PPV (95.4% vs. 85.6%), NPV (97.8% vs 92.9%) and ACC (97.4% vs. 91.8%) were obtained by BTI in comparison with MPRAGE. Good diagnostic confidence and excellent diagnostic and interreader agreements were achieved by BTI, which were superior to MPRAGE on detecting the chronic thrombus.

**Conclusion:**

BTI allows direct visualization of non-acute DVT within the dark venous lumen and has the potential to be a reliable diagnostic tool without the use of contrast medium.

## Background

Deep vein thrombosis (DVT) is a common but elusive illness, affecting about 1 in 1000 population every year [1]. Its sequelae include post-thrombotic syndrome and pulmonary embolism, which can result in long-term disability or death [2]. Accurate detection of thrombosis and assessment of its size and distribution are critical for treatment decision-making [3–6]. Due to nonspecific clinical presentations in DVT patients, the current diagnostic work-up relies heavily on objective tests including medical imaging [6, 7].

Ultrasonography (US) is a daily used technique because of its noninvasiveness, low cost and wide availability [8]. It has largely replaced the gold standard but invasive and radiation-required approach, x-ray venography, in clinical settings [7, 9]. However, the sensitivity and specificity of US are operator dependent and vary with interrogated stations, particularly at pelvic veins due to the limitation of US penetration [7, 8, 10]. Moreover, US is unable to differentiate recurrent thrombosis from residual thrombosis and thus can only provide limited guidance for DVT treatment [11–14].

As a noninvasive imaging modality with excellent soft tissue contrast, cardiovascular magnetic resonance (CMR) has increasingly gained popularity in DVT diagnostic work-up [6, 13]. Two typically used MR techniques are contrast-enhanced MR venography (CE-MRV) and three dimensional magnetization prepared rapid acquisition gradient echo (MPRAGE) [15, 16]. The former is an accurate and reliable method but unsuitable for patients with severe renal insufficiency or pregnancy due to the risk of nephrogenic systemic fibrosis (NSF) or other allergic reactions secondary to gadolinium-based contrast medium [17]; the latter is very sensitive to subacute DVT [13] but poor in detecting chronic thrombus due to similar signal intensities between the intraluminal blood and thrombus [16, 18]. Therefore, an MR technique that can diagnose DVT, especially the chronic one, without the need for contrast medium is highly desirable.

Recently, T1-weighted 3D variable flip angle turbo-spin-echo (SPACE) was proposed as a black-blood CMR approach to the detection of venous thrombosis [19, 20]. With the blood flow signal suppressed by the inherent black-blood effect of SPACE, this method can directly visualize the intraluminal thrombus. However, because the venous blood flow can be tremendously slow in deep veins, especially when severe thrombosis is present, it could still be challenging for SPACE to achieve adequate suppression of flow signal that might otherwise become a confounder for thrombus identification [19]. On the other hand, flow signal suppression in SPACE imaging can be improved by using additional black-blood preparation - delay alternating with nutation for tailored excitation (DANTE) [21–24]. Li et al. found that DANTE is capable of suppressing the signal of flow at a velocity as low as 1 mm/s [22]. Hence, we hypothesized that DANTE-prepared SPACE would be suitable for the detection of DVT.

In this work, we sought to develop a black-blood thrombus imaging (BTI) technique based on DANTE-prepared SPACE and to evaluate whether BTI could provide acceptable diagnostic performance, particularly in the non-acute DVT, while overcoming the limitations with CE-MRV and MPRAGE.

## Methods

### CMR sequence

**Fig. 1:**
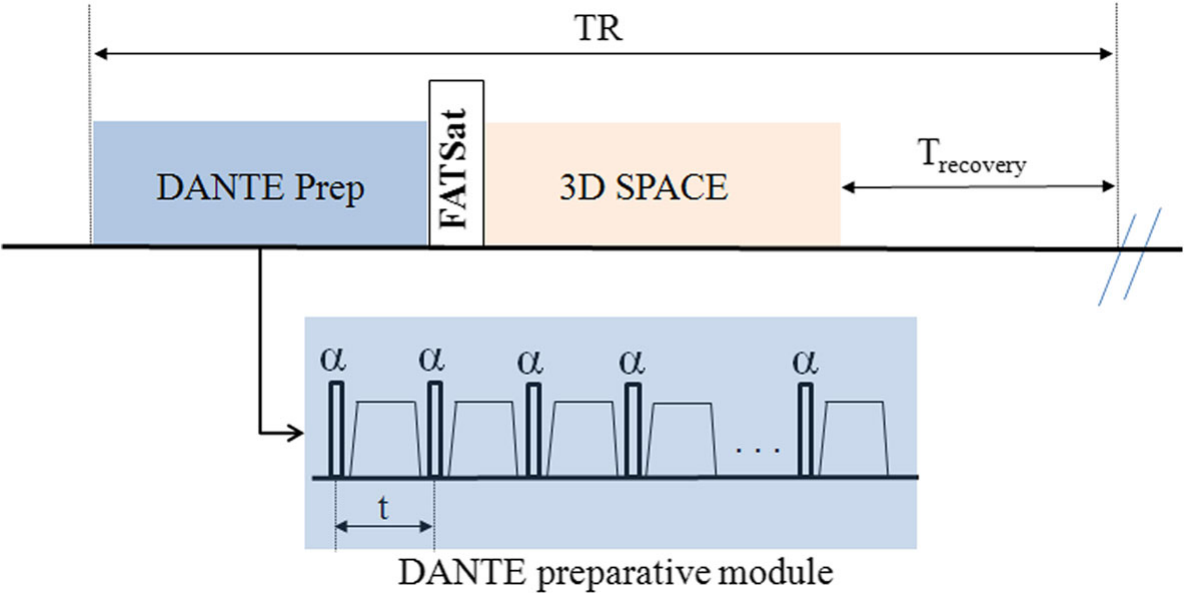
Sequence diagram of the BTI technique. The DANTE preparative module consists of a train of hard RF pulses interspersed with unipolar dephasing gradients. A fat saturation module is played out immediately before the SPACE readout to suppress fat signal

The sequence diagram of BTI is shown in Fig. 1, which consists of a DANTE black-blood preparation and a spectrally-selective fat saturation played out immediately before SPACE readout. The DANTE preparation consists of a train of hard radio-frequency (RF) pulses interspersed with unipolar dephasing gradients which are simultaneously applied on all axes. As discussed in Li et al. [22], the blood-attenuation efficiency of DANTE depends on its RF pulse flip angle, pulse train length (PTL), and zeroth gradient moment between two adjacent pulses. Previous work demonstrates that a large amount of the combinations between the RF pulse flip angle and the PTL can achieve optimal blood signal suppression. Thus, an optimal combination of these parameters is imperative to ensure satisfactory flow signal suppression. To account for the real flow characteristics in deep veins, we chose to optimize DANTE parameters through in-vivo studies. In order to simplify the parameter optimization process, the duration (0.9 ms) and strength (20 mT/m) of gradient pulses and RF pulse flip angle (15°) were kept fixed, which are within a range typically reported in literature [21, 23–26]. Thus, PTL was the only parameter to be optimized in the following healthy volunteer study. Moreover, SPACE is configured as a T1-weighted readout in favor of the detection of subacute thrombus that appears hyper-intense on T1-weighted images [27, 28].

### Subjects

Both healthy volunteer and patient studies were approved by the local institutional review board and written informed consent obtained from all participants. Eleven healthy subjects (mean age 25.3 ± 2.7 years, 6 women) who have no history of DVT were recruited for a PTL optimization study. Eighteen patients (mean age 53.9 ± 12.0 years, 7 women) with DVT diagnosed based on their clinical symptoms and US examinations within 3 days were prospectively recruited for a clinical validation study (Table 1). For safety consideration, only stable patients (including those received implanted filter at inferior vena cava) were recruited in the study. According to the time from symptom onset, these patients were classified into three groups: one subacute DVT patient (15–28 days); 11 subacute-to-chronic DVT patient (29–180 days); and 6 chronic DVT patients (>180 days). All but three patients had prior diagnosis in one leg only. Exclusion criteria for patient recruitment were known contraindications against MR imaging, known allergy to gadolinium-based contrast medium, and impaired renal function (estimated glomerular filtration rate < 30 mL/min/1.73 m^2^).

**Table 1:** Patient Characteristics

Characteristics	Values
Age, mean ± SD (range), years	53.9 ± 12.0 (35–72)
Male sex, *n* (%)	11 (61.1)
Symptoms	
Leg pain, *n* (%)	11 (61.1)
Swelling, *n* (%)	13 (72.2)
Relapsing DVT, *n* (%)	4 (22.2)
DVT Stages	
Acute stage, *n* (%)	1 (5.6)
Subacute-to-chronic stage, *n* (%)	11 (61.1)
Chronic stage, *n* (%)	6 (33.3)
MRI	
Analyzed vessel segments, *n*	304
Iliac segments, *n* (%)	42 (13.8)
Femoral segments, *n* (%)	68 (22.4)
Popliteal-crural segments, *n* (%)	194 (63.8)
Thrombotic segments with consensus reading, *n*	57
Iliac segments, *n* (%)	7 (12.3)
Femoral segments, *n* (%)	19 (33.3)
Popliteal-crural segments, *n* (%)	31 (54.4)

*SD* Standard Deviation, *DVT* Deep Vein Thrombosis

### Healthy volunteer study

Imaging was performed on a 3 T system (Siemens Tim Trio, Germany) with two standard 6-channel body coils and an integrated spine coil. Multi-slice time-of-flight was first acquired for vein localization. A 3D imaging volume with a craniocaudal spatial coverage either from the iliac to common femoral veins or from the common femoral to tibial veins was then randomly prescribed for the following optimization scans.

BTI scans were repeated with different DANTE PTL (i.e. 50, 75, 100, 125, 150, 175, 200) and the same oblique coronal imaging volume (covering both legs) and SPACE readout parameters. A conventional SPACE scan (i.e. PTL = 0) with the same imaging setting was also performed to serve as the reference for the comparison among 7 BTI scans. By this means, an optimal PTL that yielded good compromise between venous flow signal suppression and signal loss in static tissues was determined. Imaging parameters for SPACE readout are summarized in Table 2.

**Table 2:** Imaging parameters of BTI, MPRAGE and CE-MRV in the patient study

Parameters	BTI	MPRAGE	CE-MRV
Repetition time, ms	650	1500	2.8
Echo time, ms	9.8	1.7	1.6
Flip angle	variable	18	18
Fat suppression	fat saturation	water excitation	fat saturation
GRAPPA in the phase-encoding direction	2	off	3
Turbo factor	40	288	NA
Slice Partial Fourier	6/8	off	6/8
Phase Partial Fourier	off	off	6/8
Bandwidth, Hz/Pixel	710	685	685
True resolution, mm^3^	1.1 × 1.1 × (1.1–1.3)	1.3 × 1.3 × 1.3	1.1 × 1.1 × 1.2
Reconstructed resolution, mm^3^	0.55 × 0.55 × (0.55/0.65)	0.65 × 0.65 × 0.65	0.55 × 0.55 × 0.60
Field of view, mm^2^	352 × 352	340 × 340	400 × 400
Partition	208–256	288	192
Scan time per sequence	3 min 23 s–4 min 49 s	6 min 23 s	27 s
Inversion time, ms	NA	400	NA

*Note: GRAPPA* Generalized Autocalibrating Partially Parallel Acquisitions, *NA* Not Applicable

### Patient study

BTI with the PTL optimized above was further validated on clinical patients by a comparison with other clinically used 3D CMR methods - CE-MRV and MPRAGE. The three CMR techniques were successively performed in each patient. In consideration of patient comfort, efforts were made to minimize the total scan time: a) all the three techniques were performed using coronal imaging orientation to efficiently scan both legs and include affected regions with large 3D imaging volumes; b) instead of using the three-station (pelvis, thigh, and calf) approach to cover the entire lower extremities, the number of stations was flexibly chosen to focus on thrombus regions based on the prior diagnosis on the distribution of thrombus in individual patients. Detailed imaging parameters for each technique are summarized in Table 2. MPRAGE was acquired with an inversion-prepared water-excitation 3D gradient-echo pulse sequence. For CE-MRV, a 3D gradient-echo acquisition was performed once before contrast injection and repeated three times (27 s/frame) immediately when contrast medium (gadopentetate dimeglumine [Magnevist; Bayer, Germany]) arrived at the iliac artery as detected with a care bolus technique. A fixed dose of 30 mL (469.01 mg/mL) contrast medium was administered intravenously at an injection rate of 3.0 mL/s followed by 20 mL saline flush injected at the same rate.

### Image analysis

#### Healthy volunteer study

All CMR images obtained from the healthy volunteer study were loaded to a workstation (Leonardo; Siemens AG, Germany) for image analysis and review.

To determine the optimal PTL for BTI, images obtained in the eight scans of each subject were coregistered and quantitatively analyzed as follows. In the conventional SPACE (i.e. PTL = 0) image set, regions with residual blood (RB) signals in the veins were first identified by two readers in consensus (W. D. and Y. Y., radiologists with over 10 years of experience in CMR). Using a region-of-interest (ROI) method, signal intensity (SI) was then measured (G. X., with over 5 years of experience) from three locations, including the lumen region with the most severe RB, its neighboring lumen region without RB, and a surrounding signal-homogeneous vastus medialis muscle region free of image artifacts; the noise, σ_n_, was also measured as the standard deviation (SD) of SI from this muscle region [25, 29]. Apparent contrast-to-noise ratios (CNRs) were calculated for the RB versus the dark venous lumen ([SI_RB_ – SI_lumen_]/σ_n_) and for the muscle versus the dark venous lumen ([SI_muscle_ – SI_lumen_]/σ_n_). The ROIs prescribed on conventional SPACE images were propagated to the other seven BTI image sets for the same CNR analyses. Optimal PTL was finally determined based on a balance between low RB-to-lumen CNR and adequate muscle-to-lumen CNR.

Additionally, the optimized BTI and conventional SPACE were compared with regard to blood signal suppression and image artifacts (especially stripe artifact potentially caused by DANTE). The same readers blinded to subjects’ identity and imaging protocols independently assessed the randomized images. A four-point scale was used to rate the image quality on a per-subject basis: 1– poor, image with severe artifacts and/or poor venous blood signal suppression; 2– fair, image with moderate artifacts and/or fair venous blood signal suppression; 3– good, image with minimal artifacts and good venous blood signal suppression; 4– excellent, image free of artifacts and with excellent venous blood signal suppression.

#### Patient study

To avoid diagnostic bias due to the fact that imaging was focused on the affected station only, the right and left legs of each patient were separately reviewed and DVT diagnosis was made on a per-segment basis. Specifically, each 3D image set was separated into two subsets, corresponding to the two legs, using Matlab 2010 (The Mathworks Inc., Natick, MA, USA). They were then randomized and loaded to the workstation for diagnosis review. The vein system was divided into the following 13 vessel segments on each side: the common, internal, and external iliac veins; the common, superficial, and deep femoral veins; the popliteal veins; the tibiofibular trunk vein; the anterior and posterior tibial veins; the fibular veins; and the great and small saphenous veins. If vessel collateralization was identified, collateral segments were also assessed.

The same readers blinded to patients’ clinical information and imaging protocols independently made a diagnosis of DVT (presence or absence) for each venous segment on BTI, MPRAGE, and CE-MRV images, respectively. All images obtained by the same technique were reviewed at a time and reviews of different techniques were at least one-week apart to reduce memory effect. One month later, consensus reading was performed on CE-MRA images only to generate a reference for evaluating the diagnostic performance of BTI and MPRAGE. Thrombus was identified as hyper-/iso- intense signal within the dark venous lumen on BTI and as filling defects in veins on CE-MRV. On MPRAGE, thrombus was identified as bright signal within the venous lumen that could be of neutral or low signal intensity relative to background tissues. Additionally, a diagnostic confidence score on a four-point scale as in Ttreitl et al. [19] (1: poor; 4: excellent) was assigned to individual segments for each technique and then an average score was calculated for each subject.

### Statistical analysis

Statistical analysis was performed by using SPSS (version 17.0; SPSS Inc, Chicago, IL, USA). Data were presented as mean ± SD. A paired two-tailed Wilcoxon signed rank test was used for the comparison of the image quality scores between conventional SPACE and optimized BTI in the healthy volunteer study, and used for the comparison of the mean diagnostic confidence scores of each subject between BTI and MPRAGE/CE-MRV in the patient study. The segments captured concurrently by all three techniques were included for analysis. Diagnostic sensitivity (SE), specificity (SP), positive and negative predictive values (PPV and NPV), and accuracy (ACC) on a per-segment basis were determined for BTI and MPRAGE, respectively, using the consensus diagnosis of CE-MRV as the reference. The diagnostic agreement between BTI/MPRAGE and CE-MRV and the interreader diagnostic agreement for each technique were determined using Cohen’s kappa test. The agreement was rated fair (kappa value κ = 0.21–0.40), moderate (κ = 0.41–0.6), substantial (κ = 0.61–0.80), or excellent (κ > 0.80) [30]. Statistical significance was defined as *p* < 0.05.

## Results

### Healthy volunteer study

**Fig. 2:**
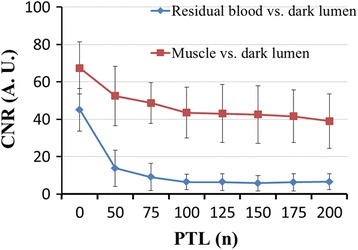
Plots of the apparent CNR versus DANTE pulse train length (PTL). DANTE with 125–175 PTL yields good black-blood effect without considerable signal loss in static tissues. Considering that the venous flow could be even slower in DVT patients, the PTL of 175 was chosen as the optimal parameter for BTI

According to CNR analysis, DANTE with a PTL of 125–175 appeared to be a suitable preparation to yield sufficient blood flow suppression without considerable signal loss in static tissues (Fig. 2). Considering that the venous flow could be even slower in DVT patients, a PTL of 175 was chosen as the optimal parameter for BTI.

**Fig. 3:**
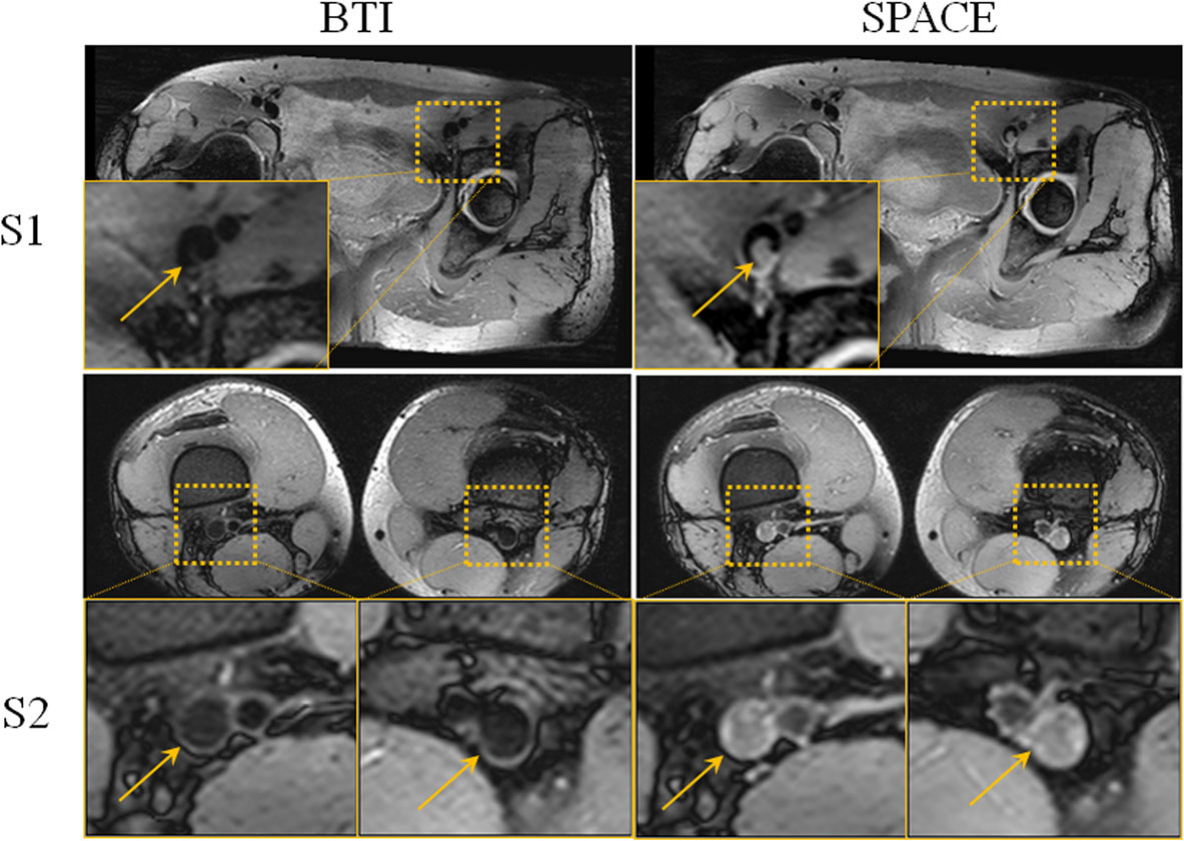
Representative images obtained from two healthy volunteers (**S1** and **S2**). Compared to SPACE, BTI more effectively nulls residual blood signals (*yellow arrows*) that would otherwise be mistaken as part of thrombus. Without the DANTE module, residual blood signals often appear in the iliac (**S1**), the distal superficial femoral and the popliteal veins (**S2**)

On conventional SPACE, residual blood signals were observed in all 11 healthy subjects and a total of 20 segments (mostly in the external iliac, distal superficial femoral and popliteal veins). This issue was substantially alleviated by using an optimal DANTE preparation, without introducing appreciable image artifacts (Fig. 3). As a result, the overall image quality score (reader 1: 3.58 ± 0.51 vs. 3.08 ± 0.51, *p* = 0.007; reader 2: 3.67 ± 0.49 vs. 2.92 ± 0.51, *p* = 0.008) was significantly higher on optimized BTI than on conventional SPACE.

### Patient study

All 18 patients successfully underwent CMR scans. In total, 304 venous vessel segments from 36 legs were assessable concurrently by scans with all three techniques and were therefore included in diagnostic performance analysis. Specifically, they were 12 common iliac, 14 internal, and 16 external iliac segments, 20 common, 28 superficial, and 20 deep femoral segments, 32 popliteal segment, 24 tibiofibular trunk segment, 24 anterior and 20 posterior tibial segments, 20 fibular segment, 34 great and 30 small saphenous segments, and 10 collateral segment.

**Fig. 4:**
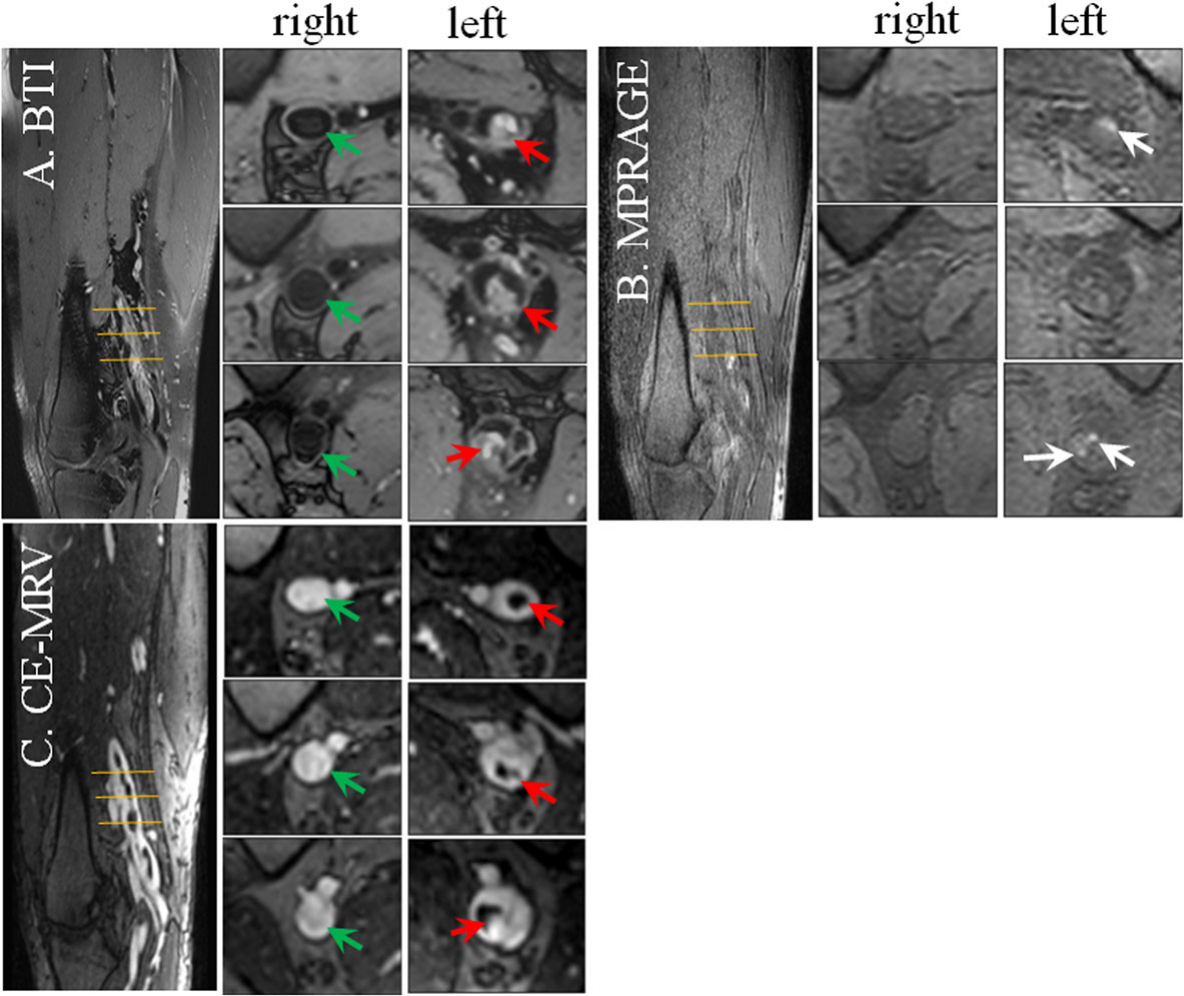
Example images obtained from a patient with chronic DVT on the left leg for 8 years. Thrombus can be detected in the distal superficial femoral and popliteal veins of the left leg by BTI and CE-MRV (*red arrows* on **a & c**). Since chronic thrombus contains less met-hemoglobin, most parts of the thrombus are moderate intensities and difficult to be differentiated from incompletely suppressed venous blood using MPRAGE (**b**). Note that a few parts of the thrombus that look like isolated islands can be seen on MPRAGE images (*white arrows* on **b**). This may be due to the resolution of DVT that fresh thrombus generates on the chronic one

**Fig. 5:**
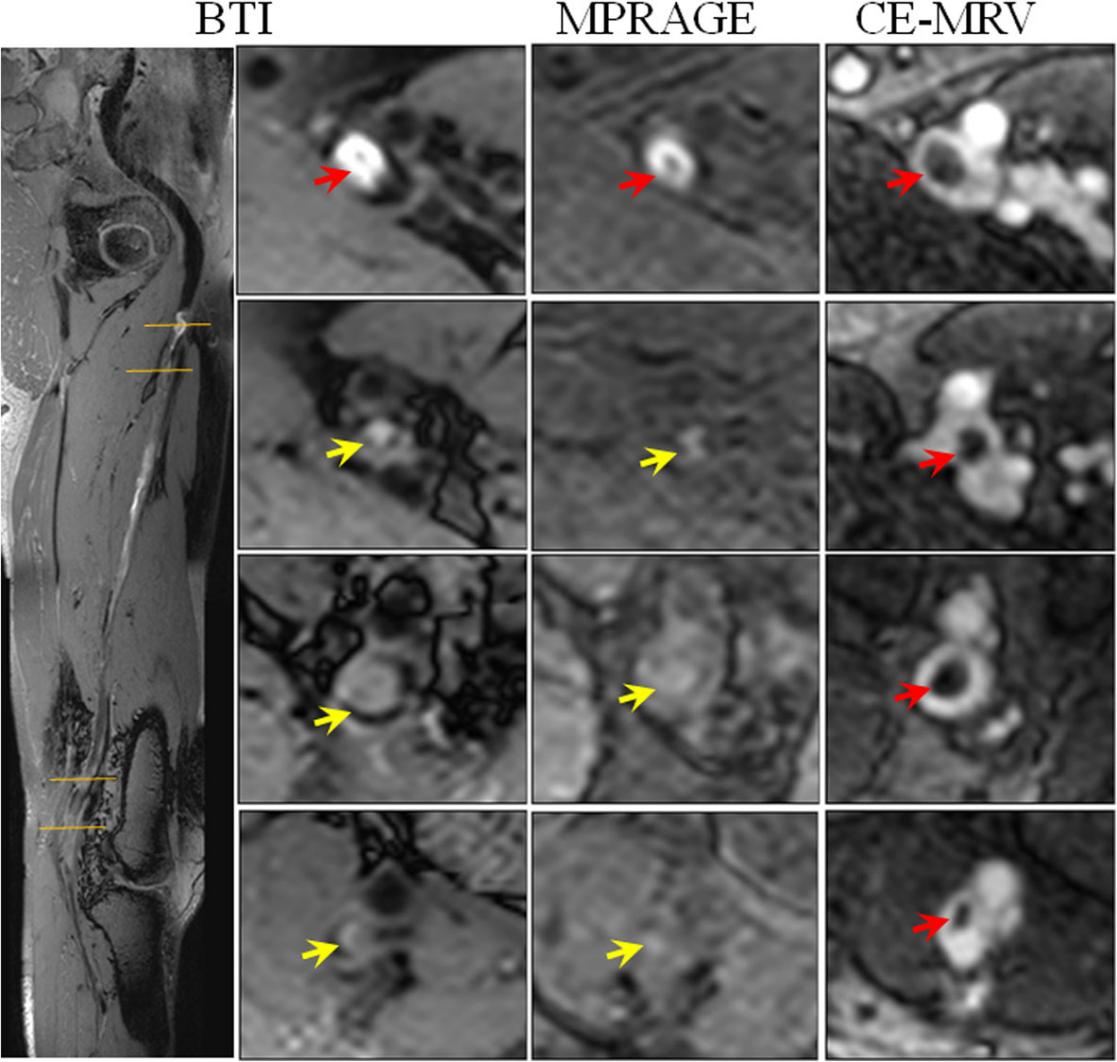
Example images obtained from a patient with subacute-to-chronic DVT on the left leg for 30 days. The thrombus detected by BTI match with those of CE-MRV. Note that parts of the thrombus appear as moderate intensities that are hardly identified by MPRAGE (*yellow arrows*). The varied intensity signals of the thrombus should correspond to its different contents of met-hemoglobin in different locations, which may reflect their stages

**Fig. 6:**
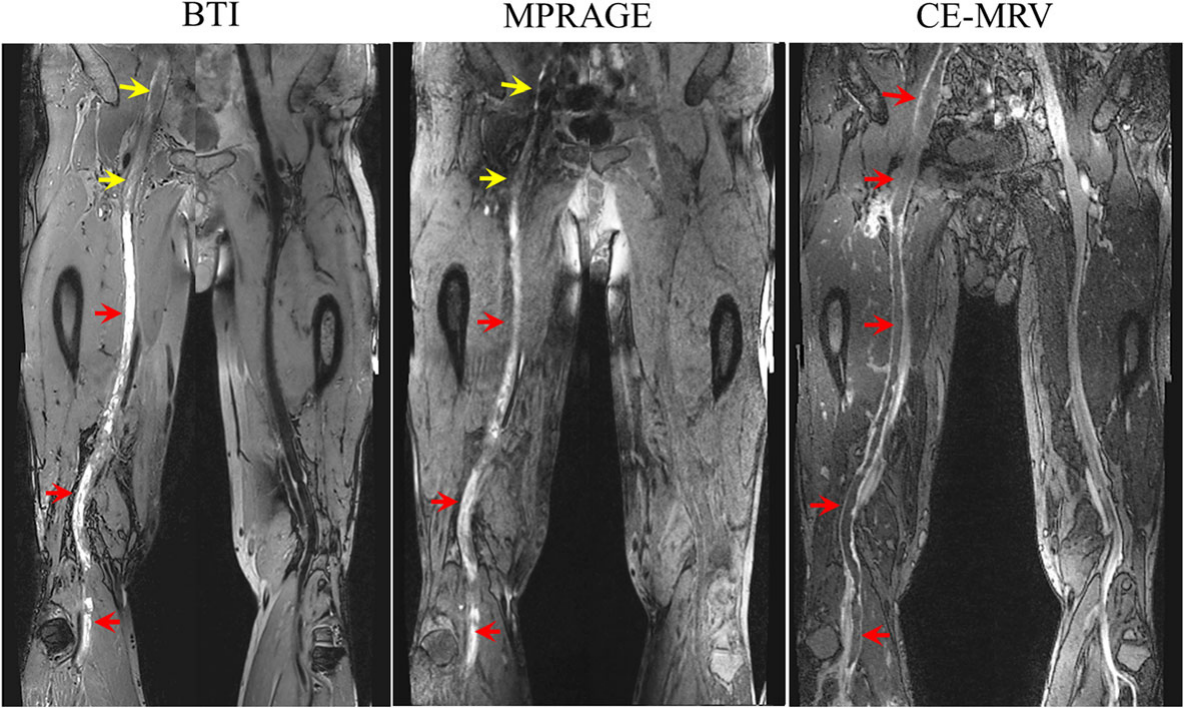
Representative curved images obtained from a patient with subacute-to-chronic DVT on the right leg for 60 days. Thrombus can be found in iliac, femoral and popliteal segments covered by two stations. Large coverage is achieved and the thrombus identified by BTI matches with that by CE-MRV. Note that parts of the thrombus appear as hyper-intense signals on BTI match with those by MPRAGE in terms of the thrombus location and shape (*red arrows*). However, other parts of the thrombus appear as iso-intense signals, which are detectable by BTI but hardly identified by MPRAGE (*yellow arrows*)

Representative images from the patient study are shown in Figs. 4, 5 and 6. In general, the thrombus detected by BTI matched with that by CE-MRV in terms of location and shape. In chronic DVT patients, thrombus was often depicted as iso-intense signals on BTI, although sporadic hyper-intense signals were also observed (Fig. 4). In subacute-to-chronic DVT patients, hyper- and iso-intense signals often coexisted on BTI, suggesting heterogeneous stages within those thrombi (Figs. 5 and 6). As expected, the hyper-intense parts of the thrombus shown on BTI were of high signal intensity and they were detectable on MPRAGE. On the other hand, the iso-intense parts of the thrombus shown on BTI were not well visualized by MPRAGE due to their similar signal intensity to the surrounding venous blood (Fig. 5). Overall, the thrombus detected by BTI matched with that by CE-MRV (Fig. 6).

The consensus reading of CE-MRV indicated thrombosis in 57 out of 304 vessel segments in all 18 patients and 21 legs (Table 3). Using this as the diagnosis reference, higher SE (average value over the two readers: 90.4% vs 67.6%), SP (average value: 99.0% vs. 97.4%), PPV (average value: 95.4% vs. 85.6%), NPV (average value: 97.8% vs 92.9%) and ACC (average value: 97.4% vs. 91.8%) were obtained by BTI in comparison with MPRAGE (Table 4). If only considering the chronic patients, much fewer thrombosis segments were correctly identified by MPRAGE compared to BTI (i.e., 6 vs. 14 by reader 1, and 8 vs. 16 by reader 2). In addition, on the per-segment level, BTI yielded excellent diagnostic agreement while MPRAGE yielded substantial one with CE-MRV (Table 4).

**Table 3:** Number of thrombosis in BTI, MPRAGE and CE-MRV

Vessel Segment		BTI reader1/reader2	MPRAGE reader1/reader2	CE-MRV with consensus reading
Iliac vessel segment	Thrombosis	7/7	6/6	7
	No thrombosis	35/35	36/36	35
Femoral vessel segment	Thrombosis	18/19	16/14	19
	No thrombosis	50/49	52/54	49
Popliteal-crural vessel segment	Thrombosis	29/28	24/24	31
	No thrombosis	165/166	170/170	163

**Table 4:** Quantitative and statistical analysis results for the comparison of BTI, MPRAGE and CE-MRV

	BTI reader1/reader2	MPRAGE reader1/reader2	CE-MRV reader1/reader2
Diagnostic confidence score, mean ± SD.	3.12 ± 0.39/3.65 ± 0.30	2.52 ± 0.27/2.16 ± 0.22	3.50 ± 0.46/3.69 ± 0.44
True positive, n	50/53	37/40	NA
True negative, n	243/246	240/241	NA
False positive, n	4/1	7/6	NA
False negative, n	7/4	20/17	NA
SE, %	87.7/93.0	64.9/70.2	NA
SP, %	98.4/99.6	97.2/97.6	NA
PPV, %	92.6/98.1	84.1/87.0	NA
NPV, %	97.2/98.4	92.3/93.4	NA
ACC, %	96.4/98.4	91.1/92.4	NA
Diagnostic agreement with CE-MRV, (κ value/*p*)	(0.88/< 0.001)/(0.95/< 0.001)	(0.68/< 0.001)/(0.73/< 0.001)	NA
Interreader agreement, (κ value/*p*)	(0.89/<0.001)	(0.64/<0.001)	(0.96/<0.001)

*Note: SD* Standard Deviation, *SE* Sensitivity, *SP* Specificity, *PPV* Positive Predictive Values, *NPV* Negative Predictive Values, *ACC* Accuracy, *NA* Not Applicable

Good diagnostic confidence scores were obtained by BTI, even though the scores were lower than those obtained by CE-MRV (average over the two readers: 3.38 ± 0.29 vs. 3.60 ± 0.43, *p* = 0.005). The scores obtained by MPRAGE (average over the two readers: 2.34 ± 0.19) were significantly lower than those obtained by BTI (*p* < 0.001) and by CE-MRV (*p* < 0.001), due to its generally lower SNR and insufficient blood signal suppression. Excellent interreader agreements on diagnosis were obtained by both BTI (κ = 0.89, *p* < 0.001) and CE-MRV (κ = 0.96, *p* < 0.001), whereas substantial interreader agreement was obtained by MPRAGE (κ = 0.64, *p* < 0.001).

## Discussion

In this work, a BTI technique was developed for the detection of DVT without the need for contrast medium. Demonstrated in a non-acute DVT patient cohort, our analysis on the per-segment basis revealed good diagnostic confidence, high SE, SP, PPV, NPV and ACC, and excellent interreader agreement, suggesting that BTI is potentially an accurate and reliable method for the diagnosis of non-acute DVT.

BTI is a 3D black-blood T1-weighted imaging technique that shares with MPRAGE some similarities but also has some differences. There is a linear relationship between the concentration of met-hemoglobin and T1 shortening of the venous thrombus [28, 31]. Because the concentrations of met-hemoglobin contained in different stages are different (i.e., low in the acute stage, high in the subacute stage, and low again in the chronic stage), the signal intensity of thrombus on T1-weighted images can be variable [28]. MPRAGE, due to its strong T1-weighted contrast, is highly sensitive to short-T1 acute to subacute thrombus. However, because the venous blood signal is usually not completely suppressed, chronic thrombus may not be distinguishable from surrounding venous blood, making it difficult to detect chronic thrombus as demonstrated in this work [16, 27]. Similarly, BTI adopts T1 contrast weighting to facilitate the diagnosis of subacute DVT that is very commonly encountered in the clinical setting. Furthermore, a black-blood contrast feature is integrated to adequately suppress venous blood signals and allow for direct visualization of thrombus even if the signal of the thrombus signal is iso-intense. This technical difference has proven to greatly facilitate sensitive and specific diagnosis of chronic thrombus and contribute to higher diagnostic confidence and reliability in this work.

Nulling the venous blood signals is crucial for BTI. While SPACE has inherent black-blood effects, residual blood signals may still be present in substantially slow venous flow which could be mistaken as part of thrombus [19]. As shown in our healthy volunteer study, without the DANTE module, residual venous blood signals often appeared in the external iliac, distal superficial, and popliteal veins using the conventional SPACE sequence. DANTE preparation features remarkably uniform signal attenuation of blood flow over a broad range of velocities above approximately 1 mm/s [22, 25]. Additionally, this approach was shown to induce less signal loss in static tissues compared to other black-blood preparation, such as motion-sensitized driven-equilibrium [32]. Thus, DANTE black-blood preparation is suitable for BTI.

BTI has the potential to be a non-contrast CMR technique for DVT diagnosis. CE-MRV is the current standard CMR technique for detection of DVT. However, gadolinium-based contrast medium is of concern in severe renal insufficiency because of the risk of NSF. Compared to CE-CMRV, BTI provides comparable diagnostic confidence level and excellent diagnostic agreement without the need for contrast medium. This makes BTI a promising non-contrast approach to DVT diagnosis, which is particularly beneficial to patients in pregnancy or with the risk of allergic reactions or NSF.

BTI is potentially useful as a DVT screening tool complimentary to US when difficult location is involved or US results are ambiguous. Fast data acquisition, high spatial resolution, and large field-of-view (FOV) in the craniocaudal direction are all important in screening DVT. The use of the SPACE readout meets the above requirements. SPACE is of a long-echo-train readout, permitting a 3D scan to be completed in a reasonable time. Furthermore, the high SNR gained by the use of both 3 T and the spin-echo-based signal acquisition allows for high spatial resolution and imaging acceleration (i.e., parallel imaging and partial Fourier). BTI of a station with an over 350 mm long craniocaudal spatial coverage and 1.1-mm isotropic resolution could be finished within 5 min. Compared with BTI, MPRAGE is also compatible with large FOV and 3 T, but comparable spatial resolution or scan time was difficult to achieve because its gradient-echo-based acquisition is relatively signal starved. There were several limitations to this study. First, imaging was focused only on the stations with DVT previously diagnosed. This is not a real clinical scenario whereby an imaging modality should be used for DVT diagnosis in the entire lower extremities. As a proof-of-principle study, this work was aimed to show whether BTI could provide acceptable diagnostic performance while overcoming the limitations with CE-MRV and MPRAGE. Hence, all the three techniques had to be performed in each patient, and imaging experiment would be prohibitively long if the entire lower extremities were assessed. Nevertheless, we have always imaged bilateral legs instead of just affected legs (15/18 patients had DVT in one leg only), and, more importantly, the right and left leg of each patient were separately reviewed in a blinded fashion and DVT diagnosis made on a per-segment basis. These measures were made to ensure sufficient “true” positive and negative samples and thus to avoid bias during image reading. A three-station imaging protocol using BTI would be more clinically useful but awaits systematic evaluation in the future. Second, the imaging parameters of MPRAGE might be suboptimal in achieving blood signal suppression. Given the TR (1500 ms) used in this study, a TI less than 450 ms would be needed but is limited by the number of partitions due to the partition-first linear reordering scheme. Increasing TR can allow for a longer TI but also make the scan even longer. Third, a small number of patients were available for each thrombus stage and, more regrettably, no acute DVT patients were investigated. Thus, the diagnostic performance of BTI for individual stages was not assessed in this work and the performance of BTI in detecting acute thrombi remains unclear. A recent study on cerebral venous thrombosis has shown the effectiveness of the SPACE sequence in depicting acute thrombi [20]. It is therefore anticipated that BTI would also be useful in acute DVT diagnosis. On the other hand, our results clearly suggest that BTI can depict thrombus with different contrast, which may potentially help stage the thrombus when combining with patient clinical history. These aspects will be investigated with a larger patient cohort in our future work. Forth, invasive X-ray venography, the gold imaging standard, was not available as a reference in our comparison study. There may be bias in the assessment of diagnostic accuracy of BTI and MPRAGE with CE-MRV as the reference. This reflects the fact that the venography is performed when the presence/absence of DVT cannot be confirmed with other noninvasive techniques [6].

## Conclusions

BTI offers direct visualization of thrombus within the dark lumen. Our results suggest that the technique is well agreement with CE-MRV in detecting non-acute DVT and good for diagnosis in the regime in which MPRAGE is known to be poor. The technique has the potential to be a quick, safe and reliable tool for the assessment of non-acute DVT without the use of contrast medium.

## Abbreviations

3D: Three dimensional
ACC: Accuracy
BTI: Black-blood thrombus imaging
CE-MRV: Contrast-enhanced magnetic resonance venography
CMR: Cardiovascular Magnetic Resonance
CNR: Contrast-to-noise ratios
DANTE: Delay alternating with nutation for tailored excitation
DVT: Deep vein thrombosis
FOV: Field-of-view
MPRAGE: Three dimensional magnetization prepared rapid acquisition gradient echo
NPV: Negative predictive values
NSF: Nephrogenic systemic fibrosis
PPV: Positive predictive values
PTL: Pulse train length
RB: Residual blood
RF: Radio-frequency
ROI: Region-of-interest
SD: Standard deviation
SE: Sensitivity
SI: Signal intensity
SP: Specificity
SPACE: Three dimensional variable flip angle turbo-spin-echo
US: Ultrasonography
